# The severity of glomerular endothelial cell injury is associated with infiltrating macrophage heterogeneity in endocapillary proliferative glomerulonephritis

**DOI:** 10.1038/s41598-021-92655-5

**Published:** 2021-06-25

**Authors:** Momoko Arai, Akiko Mii, Tetsuya Kashiwagi, Akira Shimizu, Yukinao Sakai

**Affiliations:** 1 Department of Nephrology, Nippon Medical School, 1-1-5, Sendagi, Bunkyo-ku, Tokyo, 113-8602 Japan; 2 Department of Analytic Human Pathology, Nippon Medical School, Tokyo, Japan

**Keywords:** Kidney diseases, Inflammation, Glomerular diseases, Nephritis

## Abstract

Endocapillary proliferation occurs in various types of glomerulonephritis (GN), with varying prognoses. We examined 42 renal biopsy samples representing endocapillary proliferative lesions from post-streptococcal acute GN (PSAGN), Henoch–Schönlein purpura nephritis (HSPN), and lupus nephritis (LN). In PSAGN, the glomerular capillary network was maintained, although severe lesions displayed dots or short, curved lines, indicating CD34-positive capillaries and suggesting capillary obstruction. Conversely, patients with LN and HSPN displayed obstruction of CD34-positive capillaries with dissociation from the glomerular basement membrane even in mild lesions. According to computer-assisted morphologic analysis, the cell density did not differ between the diseases. However, in PSAGN, the number of capillary loops was significantly increased, with a larger glomerular capillary luminal area than in the other groups. In addition, the number and frequency of CD163-positive cells (M2 macrophages) tended to be higher in PSAGN, while there were no significant differences in the number of CD68-positive (total) macrophages. These results indicate that in PSAGN, endothelial cell damage is less severe, and angiogenesis may be promoted. The severity of endothelial cell injury in each disease may be associated with differences in infiltrating inflammatory cell phenotypes.

## Introduction

Endocapillary proliferation is characterized by the proliferation of resident glomerular cells, such as mesangial and endothelial cells, together with leukocyte infiltration of the glomeruli, resulting in narrowing and occlusion of the glomerular capillary lumina^1^. According to the International Society of Nephrology and the Renal Pathology Society (ISN/RPS) classification of lupus nephritis (LN), endocapillary proliferative (EP) lesions are considered active lesions^2,3^. EP lesions are also considered active lesions as per the Oxford classification of IgA nephropathy^4^. Furthermore, a recent Japanese study reported that EP lesions might be a prognostic factor for IgA nephropathy^5,6^. Conversely, in post-streptococcal acute glomerulonephritis (PSAGN), EP lesions are a typical pathological feature, and are considered transient lesions with generally favorable prognosis.

Although EP lesions are observed in various glomerular diseases, different forms of glomerulonephritis (GN) with EP lesions have different prognoses. Therefore, we hypothesized that there might be qualitative differences between these EP lesions, which appear identical by light microscopy. Wu et al. reported differences in the levels of various cell types in EP lesions, including both infiltrating and resident glomerular cells, suggesting that these lesions have different characteristics^7^. In clinical practice, EP lesions in HSPN and LN sometimes progress to crescent formations with rupture of the glomerular basement membrane (GBM), contrary to PSAGN. We previously reported the loss of endothelial cell-positive glomerular capillaries in extracapillary proliferative lesions (using CD34 as a marker of endothelial cells)^8^. We suggested that severe endothelial cell injury is associated with crescent formation and poor prognosis in the active phases of HSPN and LN^8^. In addition, macrophages are thought to play important roles in various forms of GN^9^. We have previously investigated macrophage heterogeneity using an experimental GN model^10,11^. These studies identified that augmentation of anti-inflammatory macrophages attenuated crescentic GN in rats. Therefore, in the present study, we examined whether glomerular endothelial cell injury severity and infiltrating macrophage subtypes are associated with differences in the prognoses of various forms of GN with EP lesions.

## Results

### Alteration of the glomerular capillary network by endocapillary proliferation

We focused on three glomerular diseases with EP lesions: PSAGN, HSPN, and LN. Patients with minor glomerular abnormalities (MGAs) were examined as controls. There were no significant differences in age, sex, or serum creatinine levels between the groups of patients (Table 1).

**Table 1:** Clinical characteristics.

	PSAGN	HSPN	LN	Control	*P*-value
Age (years)	20.5 [11.5–31]	23 [16.8–51]	30.5 [11.3–46.8]	22.5 [15.8–38.3]	n.s.
Sex (F/M)	6/6	6/6	12/6	3/5	n.s.
sCre (mg/dL)	0.915 [0.73–1.14]	0.63 [0.51–0.89]	1.345 [0.57–1.58]	0.77 [0.64–0.87]	n.s.

PSAGN, post-streptococcal acute glomerulonephritis; HSPN, Henoch–Schönlein purpura nephritis; LN, lupus nephritis; sCre, serum creatinine level; n.s., not significant.

**Figure 1:**
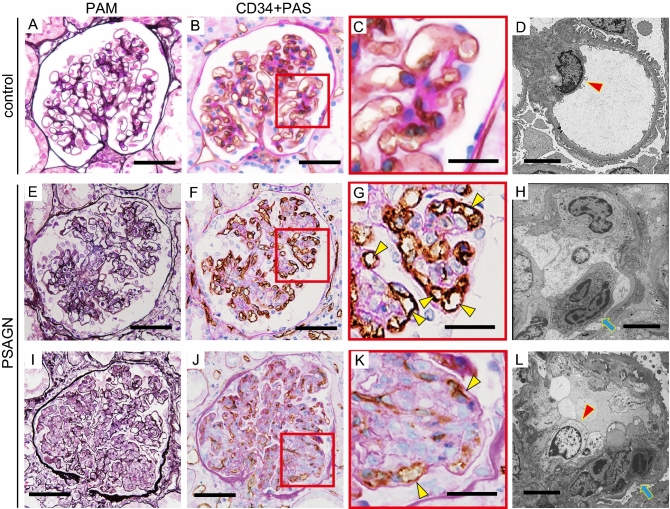
Alteration of the glomerular capillary network by endocapillary proliferation. Representative images of control samples (**A**–**D**) and PSAGN with mild (**E**–**H**) and severe (**I**–**L**) EP lesions. (**A**,**E**,**I**) show PAM staining; (**B**,**C**,**F**,**G**,**J**,**K**) show CD34 staining with PAS counterstain; and (**D**,**H**,**L**) show electron microscopy. Arrowheads indicate endothelial cells (**D**,**L**). Arrows indicate inflammatory cells (**H**,**L**). Scale bars, 50 μm (**A**,**B**,**E**,**F**,**I**,**J**); 20 μm (**C**,**G**,**K**); 5 μm (**D**,**H**,**L**).

In patients with MGAs, light microscopy images of periodic acid silver methenamine (PAM)-stained samples displayed normal glomeruli, without increased cell proliferation, extracellular matrix accumulation, or glomerular deposits (Fig. 1A). To explore the glomerular capillary network, we immunostained serial sections for CD31, CD34, and von Willebrand factor (vWF), which is expressed on the endothelial cell surface and periodic acid-Schiff (PAS) counterstain (Supplementary Figure 1A–C). Immunostaining for CD31 and vWF was weak and undetectable compared to CD34 staining. As we have used CD34 immunostaining to detect glomerular endothelial cells in healthy and diseased glomeruli, we used this method here as well^8,12,13^. Glomerular capillary loops were circular and well dilated, which implied normal glomerular blood flow in patients with MGAs (Fig. 1B,C). Electron microscopy (EM) images showed endothelial cells with maintained fenestra (Fig. 1D). Conversely, in patients with PSAGN with mild endocapillary proliferation (Fig. 1E), the number of small capillary loops defined by CD34-positive endothelial cells increased, and they were relocalized to the periphery of the glomerular tufts (Fig. 1F,G). Interestingly, these capillaries were not collapsed. By EM, we observed endothelial cell swelling and disappearance of the fenestra, as well as inflammatory cell infiltration into the glomerular capillaries (Fig. 1H), indicating glomerular endothelial cell injury. In severe EP lesions, CD34-positive areas displayed dots or short curved lines, indicating collapsed capillaries (Fig. 1I,J). High-magnification images revealed that CD34-positive endothelial cells were separated from the GBM, suggesting a widening of the subendothelial spaces (Fig. 1K). EM images clearly showed the migration of inflammatory cells into the subendothelial spaces and the swelling of endothelial cells (Fig. 1L). However, the GBM structure was maintained, and fibrin exudation was rarely observed.

### Endocapillary proliferation results in severe glomerular capillary injury in HSPN and LN

**Figure 2:**
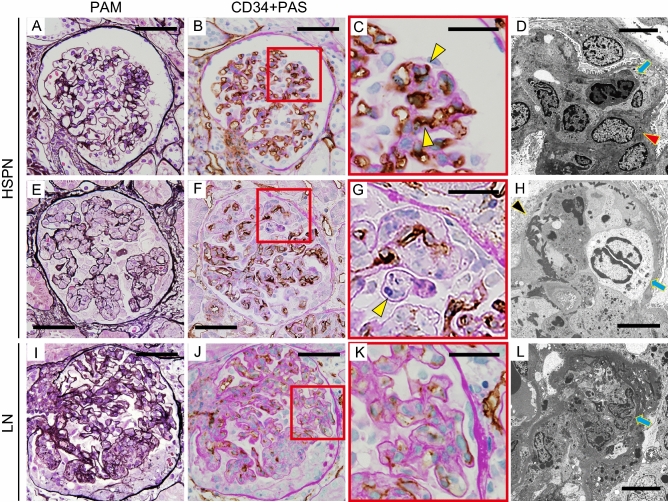
Severe glomerular capillary injury by endocapillary proliferation in HSPN and LN. Representative images of moderate (**A**–**D**) and severe (**E**–**H**) HSPN lesions and severe LN lesions (**I**–**L**). (**A**,**E**,**I**) show PAM staining; (**B**,**C**,**F**,**G**,**J**,**K**) show CD34 staining; and (**D**,**H**,**L**) show electron microscopy. Arrowheads (black) indicate GBM thinning (**H**). Arrowheads (red) indicate endothelial cells (**D**). Arrows indicate inflammatory cells (**D**,**H**,**L**). Scale bars, 50 μm (**A**,**B**,**E**,**F**,**I**,**J**); 20 μm (**C**,**G**,**K**); 5 μm (**D**,**H**,**L**).

We next assessed changes in the glomerular capillary network by PAM staining and CD34 immunostaining of samples from patients with HSPN and LN with EP lesions. In patients with HSPN with mild segmental EP lesions (Fig. 2A), CD34-positive glomerular capillary loops were narrowed and occasionally separated from the GBM (Fig. 2B,C, arrowheads), unlike in PSAGN. EM images showed endothelial cell swelling and loss of fenestra, and migration of inflammatory cells into the subendothelial space despite only mild proliferation (Fig. 2D). In moderate and severe global proliferative lesions (Fig. 2E), CD34-positive areas presented dots or curved lines, and some disappeared (Fig. 2F,G, arrowhead). EM revealed infiltration by numerous inflammatory cells, the disappearance of endothelial cells with massive fibrin exudation, and thinning of the GBM (Fig. 2H).

Similar to HSPN, samples from patients with LN also displayed narrowing and obstruction of CD34-positive capillaries and dissociation from the GBM, even in mild EP lesions (Fig. 2I–L). In some lesions, CD34 staining was very weak (Fig. 2K). In severe global proliferative lesions, CD34-positive cells were expectedly lost, and cellular crescents formed (Fig. 2J). EM also showed severe endocapillary hypercellularity and the loss of endothelial cells with fibrin exudation. These findings indicate severe endothelial cell injury (Fig. 2L).

### Associations between disease activity and glomerular endothelial cell injury severity in GN with EP lesions

**Figure 3:**
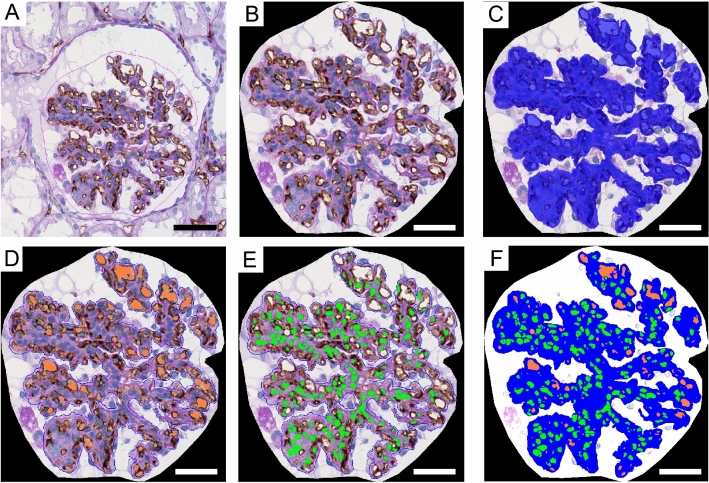
Morphometric analysis process using a computer-assisted image analyzer. Morphometric analysis performed on a PSAGN biopsy sample that has been immunostained for CD34 with PAS counterstain. The glomerulus is outlined in pink (**A**). The outline of the glomerular tuft was extracted (**B**) and the glomerular tuft area was colored blue (**C**). The capillary lumen area was colored orange (**D**). The nuclei were colored green (**E**). These images were combined (**F**). Scale bars, 50 μm (**A**); 30 μm (**B**–**F**).

All patients with LN were diagnosed as Class IV (ISN/RPS) with active lesions. Histological findings showed that patients with HSPN and LN had significantly higher incidences of extracapillary proliferative lesions (i.e., crescent formation rates) than those with PSAGN (Table 2). The histological findings indicate that disease activity differs between PSAGN and the two other diseases. Based on the CD34 immunostaining results, we hypothesized that there might be differences in the severity of endothelial cell damage in EP lesions between the diseases. Therefore, we performed a quantitative analysis of their morphologies using computer-assisted morphometric analysis. Details of image analysis are described in “Methods” section (Fig. 3A–F). First, computer processing of the images of CD34 and periodic acid-Schiff (PAS) combined staining demarked the area of capillary loops and the nuclei of cells in a glomerular tuft, colored orange and green, respectively (Fig. 3D,E). The total glomerular tuft area is the sum of the orange, green, and blue areas (Fig. 3F). These images clarified the area of the glomerular capillaries and the cell number in the glomerular tuft.

**Table 2:** Pathological characteristics.

	PSAGN	HSPN	LN	*P*-value
Crescent formation rate (%)	0 [0–0]	11.67 [7.36–17.8]	9.72 [0–21.5]	< 0.05
Interstitial fibrosis (%)	0 [0–6.25]	5 [0–7.5]	5 [0–20]	n.s.
Sclerotic glomeruli (*n*)	0 [0–3.84]	6.67 [0–18.6]	0 [0–3.45]	n.s.
Arteriosclerosis score	0 [0–1]	0.5 [0–1]	1 [0–1]	n.s.

PSAGN, post-streptococcal acute glomerulonephritis; HSPN, Henoch–Schönlein purpura nephritis; LN, lupus nephritis; n.s., not significant.

**Figure 4:**
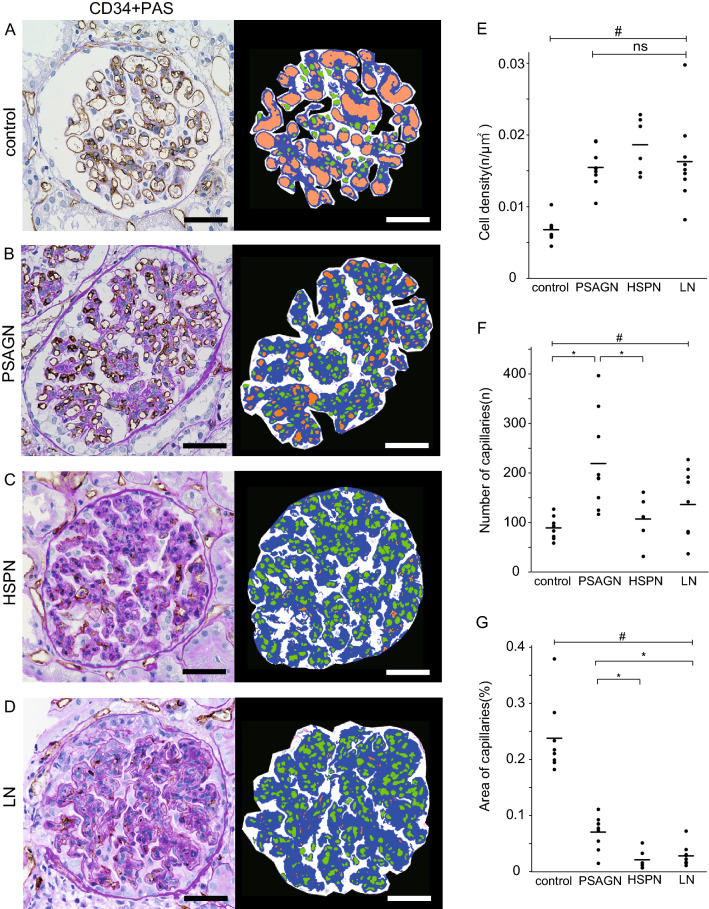
Association between disease activity and glomerular endothelial cell injury in GN with EP lesions. Representative images of control (**A**), PSAGN (**B**), HSPN (**C**), and LN (**D**) samples. The left panels of (**A**,**B**,**C**,**D**) show CD34 immunostaining with PAS counterstain, and right panels show images from the computer-assisted image analysis of same glomeruli. The nuclei and capillary lumen are shown in green and orange, respectively. The total glomerular tuft area is the sum of the orange, green, and blue parts. Graphs show the cell density (**E**), number of capillaries (**F**), and percentage of capillary lumen area per total glomerular tuft area (**G**). Scale bars, 50 μm. ^#^*p* < 0.05 among the four diseases by Kruskal–Wallis test, *p < 0.05 between each group by Steel–Dwass test, ns, no significance.

In the control group, the capillary luminal area (orange) was large (Fig. 4A). In PSAGN, many tiny orange circles were observed in the glomerular tuft; however, the orange area was small (Fig. 4B). Conversely, among HSPN and LN groups, the orange area was barely detected (Fig. 4C,D). This indicates the obstruction or loss of the glomerular capillaries by severe glomerular endothelial injury. Notably, as expected, the cell number was increased in all GN groups compared with the control group (Fig. 4A–D). Next, we quantitatively assessed glomerular cell density, the number of capillary loops, and the area of the glomerular capillary lumina (Fig. 4E–G). As expected, glomerular cell density was significantly higher in all three diseases with EP lesions compared to the control group (Fig. 4E). Interestingly, the number of capillary loops was remarkably increased in PSAGN (Fig. 4F), although there were no significant differences in glomerular cell density among the three diseases (Fig. 4E). Furthermore, the glomerular capillary lumina area was larger in PSAGN than in HSPN and LN (Fig. 4G). This indicates that endothelial cell damage is relatively benign in PSAGN compared to the other two diseases. The increased number of small capillaries in PSAGN and the loss of capillaries in HSPN/LN may have occurred in response to direct endothelial damage or the glomerular inflammatory milieu. In addition, we confirmed cells-double positive for CD34 and Ki67 in the glomeruli, indicating proliferating glomerular endothelial cells (Supplementary Figure 2).

### Characteristics of infiltrating cells in GN with EP lesions

**Figure 5:**
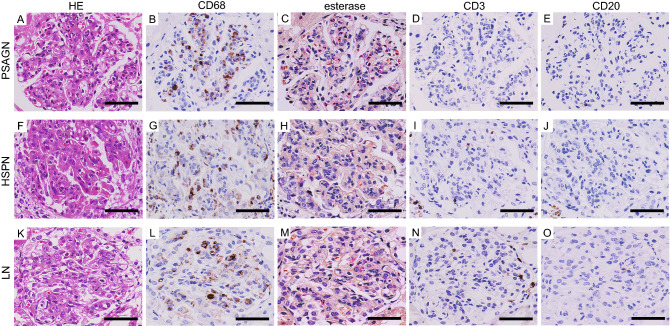
Characteristics of infiltrating cells in GN with EP lesions. Representative images of PSAGN (**A**–**E**), HSPN (**F**–**J**), and LN (**K**–**O**). HE staining (panels A, F, and K) and immunostaining for CD68 (a pan-macrophage marker; **B**,**G**,**L**), esterase (a neutrophil marker; **C**,**H**,**M**), CD3 (a T lymphocyte marker; **D**,**I**,**N**), and CD20 (a B-lymphocyte marker; **E**,**J**,**O**). Scale bars, 50 μm.

We hypothesized that the difference in the endothelial cell injury severity between PSAGN and HSPN/LN might be due to differences in infiltrating inflammatory cell characteristics. Therefore, we next evaluated the type of glomerular inflammatory cells in each disease (Fig. 5A–O). We performed additional staining for hematoxylin and eosin (HE; Fig. 5A,F,K), CD68 (a pan-macrophage marker; Fig. 5B,G,L), esterase (a neutrophil marker; Fig. 5C,H,M), CD3 (a T lymphocyte marker; Fig. 5D,I,N), and CD20 (a B lymphocyte marker; Fig. 5E,J,O), using serial sections. These cases presented a similar degree of hypercellularity with inflammatory cell infiltration. Abundant CD68 positive cells infiltrated the glomeruli in all three groups, while neutrophils were sparse and a few CD3 and CD20-positive lymphocytes were observed. We used tonsil tissue to verify the reliability of the antibodies against CD3 and CD20 (Supplementary Figure 3).

### Heterogeneity of infiltrating macrophages in GN with EP lesions

**Figure 6:**
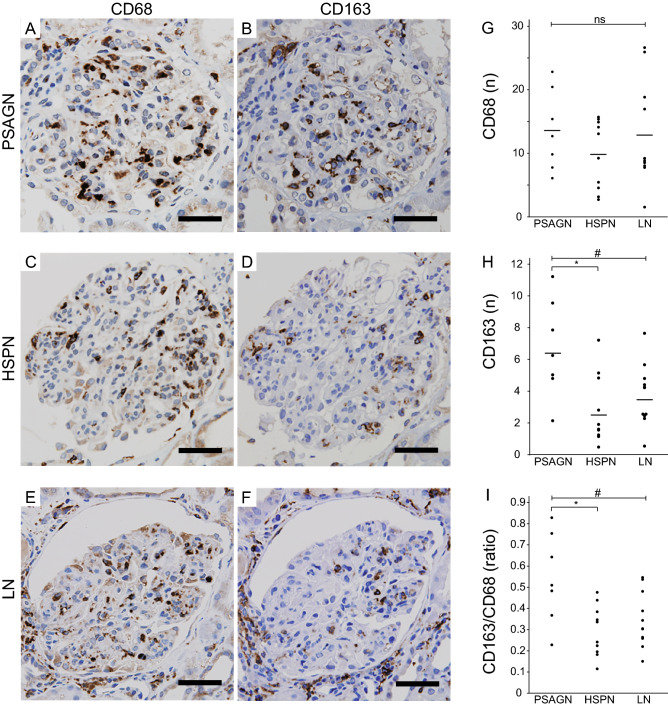
Heterogeneity of infiltrating macrophages in GN with EP lesions. CD68 and CD163 immunostaining of PSAGN (**A**,**B**), HSPN (**C**,**D**), and LN (**E**,**F**). Graphs show the number of cells positive for CD68 (**G**) and CD163 (**H**) and the frequency of CD163-positive cells per CD68-positive cell (**I**). Scale bars, 50 μm. ^#^*p* < 0.05 among the three diseases by Kruskal–Wallis test, *p < 0.05 between each group by Steel–Dwass test, ns, no significance.

Since a large number of CD68-positive cells infiltrated the EP lesions, we next focused on macrophage heterogeneity. To determine the tissue distribution of macrophages in the glomerulus, we performed double immunostaining for type IV collagen and CD68 (Supplementary Figure 4). In PSAGN, CD68-positive cells were distributed mainly in the capillary lumina, while in the other two groups, CD68-positive cells infiltrated into the mesangial and subendothelial areas as well as the capillary lumina. Next, we performed CD163 immunostaining (an M2 macrophage marker; Fig. 6A–F) in addition to CD68 and performed a quantitative analysis. Surprisingly, there were no significant differences in the numbers of CD68-positive macrophages between the three diseases (Fig. 6G), while the number of CD163-positive cells and the ratio of CD163/CD68-positive cells were significantly higher in PSAGN than in HSPN and LN (Fig. 6H,I). In pairwise comparisons, the number of CD163-positive macrophages were significantly higher in the PSAGN group than HSPN group. On the other hand, CD206-positive cells (an M2a macrophage marker) in the glomeruli tended to be more prevalent in LN cases than the other groups although the number of samples was not sufficient for analysis (Supplementary Figure 5).

## Discussion

In the present study, we analyzed qualitative differences in the EP lesions in PSAGN, HSPN, and LN, focusing on the severity of endothelial cell injury. CD34 immunohistochemical staining is useful to detect the glomerular capillary network^7,8,12^. CD34 has been mainly reported to detect endothelial cells^14–16^; however, it is also expressed on the surface of lymphohematopoietic stem and progenitor cells, bone marrow stromal cells, and embryonic and immature fibroblasts^14–16^. Some reports indicate that CD34 may also be expressed in mesangial cells^17,18^. Therefore, we stained for additional endothelial cell markers (CD31 and vWF), which confirmed that CD34 immunostaining clearly identified glomerular endothelial cells in the cases of our study.

In PSAGN samples, although the small loops of CD34-positive glomerular capillaries were relegated to the periphery of glomerular tufts, many capillary lumina remained circular and were only lost in severe lesions. Conversely, in HSPN and LN, CD34-positive capillaries were narrowed and obstructed even in mild lesions, often displaying dots or curved lines indicating capillary collapse. In some lesions, loss of CD34-positivity was observed. These findings were visualized and successfully quantified by measuring the glomerular cell density and the number and area of the glomerular capillaries. Glomerular cell density was significantly increased in all GN groups compared to the control group. There were no significant differences among the three groups, which displayed similar levels of cell proliferation. Interestingly, the number of capillary loops was significantly increased in PSAGN samples only, while HSPN and LN samples showed no differences relative to control samples. These results indicate the following regarding PSAGN: first, the glomerular capillary network was retained, as endothelial cell injury was less severe. Second, glomerular repair was actively promoted; therefore, the number of glomerular capillaries was markedly increased. We presume that there may be disease specificity in both the severity of endothelial cell injury and the subsequent repair processes that occur.

HSPN is a typical form of small-vessel vasculitis that targets the glomerular capillaries. A previous semi-quantitative analysis using a marker for glomerular endothelial cells reported particularly severe endothelial cell injury in HSPN among several types of GN^19^. The pathology of HSPN is characterized by vascular damage mediated by an immune response to galactose-deficient immunoglobulin A (IgA)1. In addition, the direct involvement of endothelial cell damage caused by IgA anti-endothelial cell antibodies (AECAs) has been reported, and has also been detected in other forms of systemic vasculitis, such as LN and antineutrophil cytoplasmic antibody-associated vasculitis^10,11,20^.

LN is generally initiated by the glomerular deposition of immune complexes (ICs), including various autoantibodies. Especially in patients categorized as class III and IV (ISN/RPS classification), subendothelial IC deposits induce glomerular endothelial damage and contribute to the development of proliferative GN^21^. AECAs have also been reported to cause direct injury to endothelial cells in LN without IC involvement^20^. Recent reports have indicated that anti-angiogenic signaling via interferon α may lead to endothelial damage, resulting in vascular repair inhibition^22,23^.

Like HSPN and LN, PSAGN is also an IC-type nephritis. It has been suggested that two antigenic fractions of *Streptococcus*, characterized by nephritis-associated streptococcal plasmin receptor and streptococcal pyrogenic exotoxin B, respectively, are related to disease induction. Most pediatric cases progress through a transient course with good prognosis. Generally, PSAGN is considered a reversible form of GN, which can probably be attributed to appropriate repair after injury^24^. In the acute phase of PSAGN, Oda et al. observed not only inflammatory cell infiltration but also the active proliferation of glomerular resident endothelial and mesangial cells, resulting in marked glomerular hypercellularity. Furthermore, they reported that apoptosis plays an important role in the repair process^25^. Our previous study using the Thy-1 nephritis model (which is reversible) revealed endothelial cell proliferation and angiogenesis in damaged glomeruli^26^. Angiogenesis is considered essential for adequate tissue repair. In our PSAGN samples, an increased number of CD34-positive endothelial cells was observed. Furthermore, cells double-positive for CD34 and Ki67 were observed, indicating that they may be undergoing angiogenesis.

Previously, we reported that severe glomerular endothelial cell injury could lead to extracapillary proliferative lesions^8^. In the present study, patients with HSPN and LN had significantly higher rates of crescent formation compared to those with PSAGN, indicating the existence of severe endothelial cell injury in HSPN/LN. Furthermore, we identified differences in the severity of glomerular endothelial cell damage using computer-assisted morphological analysis.

Generally, the infiltration of many inflammatory cells is observed in EP lesions, in addition to the increase in resident glomerular cells. We confirmed that abundant macrophages infiltrated EP lesions in all diseases. A previous report also reported increased macrophages in GN with EP lesions^7^. Macrophages play important roles in various forms of GN^9^. They are classified into two functional subtypes: M1 and M2. M1 macrophages are proinflammatory, are activated via the classical pathway, and function in phagocytosis and oxidative injury, while M2 macrophages are anti-inflammatory, are activated via the alternative pathway, and function in tissue repair, fibrosis, and apoptosis^9,27,28^. There are several subtypes of M2 macrophages. CD163 is a typical marker of M2c macrophages, which are associated with anti-inflammatory effects. A previous report suggested that the anti-inflammatory role of CD163-positive macrophages is characterized by the production of IL10^29^. Our study revealed that the number and frequency of infiltrating CD163-positive macrophages were significantly higher in PSAGN among three groups, while there were no significant differences in the number of total macrophages (indicated by the pan-macrophage marker KP1) among the three groups. In LN, the number and frequency of infiltrating CD163-positive macrophages tended to be lower than in PSAGN; however, the difference was not statistically significant. Another report also reported significantly increased M2 macrophage infiltration into the glomeruli in patients with class III and IV (ISN/RPS classification) LN^30^. Functional abnormalities in M2 macrophages have been suggested in LN^30,31^. In our LN cases, greater numbers of CD206-positive cells (M2a macrophages) were observed, while few were found in PSAGN and HSPN glomeruli. It has been previously reported that CD206-positive macrophages are abundant in renal tissue in LN and are associated with its pathogenesis. On the other hand, our results suggested that CD163-positive macrophages are more common in PSAGN and contribute to its benign phenotype. Taken together, the results indicate that the severity of endothelial cell injury in GN depends on the disease and may be dictated by the different phenotypes of infiltrating cells. Further studies will be required to identify the factors concerned with repairing and maintaining capillaries after injury and those regulating the proliferation of resident glomerular cells. These factors could represent new therapeutic targets for various glomerular diseases.

## Methods

### Ethics statement

The study protocol was performed according to the Declaration of Helsinki and was approved by the Human Ethics Committee of Nippon Medical School (approval number, B-2020-167). Renal biopsies were performed with informed consent. Informed consent was obtained from all patients or, if patients are under 18, from their parents or legal guardians.

### Case selection and clinical findings

Kidney biopsy tissues obtained from patients in the Department of Pathology at Nippon Medical School between 1998 and 2014 were retrospectively assessed. We selected 50 cases presenting with EP lesions due to PSGN (*n* = 12), HSPN (*n* = 12), and LN (*n* = 18). For the control group, we selected eight patients diagnosed with MGA by kidney biopsy. The ages, sexes, and serum creatinine levels (mg/dL) of the patients at the time of biopsy were retrospectively examined using clinical records.

### Pathological examination

We examined kidney biopsies with light microscopy, immunohistochemistry, fluorescence microscopy, and electron microscopy. For light microscopy, we prepared sections from formalin‐fixed paraffin‐embedded (FFPE) tissue, stained them with PAM stain and following markers. To identify endothelial cells, we immunoassayed the samples for CD31 (cat. no. M0823, DAKO), CD34 (cat. no. NU-4A1; Nichirei Bioscience) and vWF (cat. no. A0082, DAKO), with PAS counterstain. To characterize glomerular infiltrating cells, we immunostained the samples for CD68 (cat. no. M0814, DAKO; a pan-macrophage marker), esterase (a neutrophil marker), CD3 (cat. no. IR503, DAKO; a T cell marker), CD20 (cat. no. M0755, DAKO; a B cell marker), CD163 (cat. no. 10D6, Novocastra; an M2 macrophage marker), CD206 (cat. no. 24595, Cell Signaling; an M2a macrophage marker), and Ki67 (cat. no. 418071, Nichirei Bioscience). To detect the locations of infiltrating macrophages, we double-immunostained the frozen samples for type IV collagen (cat. no. 1340-01, Southern Biotechnology, a marker for basement membrane) and CD68. EM was performed on tissues fixed in 2.5% glutaraldehyde, post-fixed in 1% osmium tetroxide, and embedded in Epon. Ultrathin sections were stained with uranyl acetate and lead citrate and then examined on a Hitachi H7500 Transmission Electron Microscope (Hitachi, Ibaraki, Japan). We evaluated the crescent formation rate ((glomeruli with crescents/all glomeruli) × 100; %), the extent of interstitial fibrosis (%), the number of sclerotic glomeruli, the atherosclerosis score (negative: 0, mild: 1, moderate: 2, severe: 3), the number of CD68+ macrophages, and the frequency of CD163+ macrophages in the glomerulus ((number of CD163+ macrophages/number of CD68+ macrophages) × 100; %).

### Computer-assisted morphometric analysis

We analyzed cases including more than five glomeruli without sclerotic lesion or crescent formation, and determined the average for each case. Using a computer-assisted image analyzer (BZ X800; Keyence, Osaka, Japan), we assessed the areas of the glomerular capillaries and the glomerular tuft. First, a full glomerular image was captured (Fig. 3A). The outline of the glomerular tuft was extracted (Fig. 3B). The glomerular tuft was roughly outlined as the blue area automatically, then glomerular epithelial cells were manually excluded (Fig. 3C). The white area enclosed by CD34-positive endothelial cells, namely the glomerular capillary area, was detected automatically and colored orange (Fig. 3D). The nuclei were detected automatically and colored green (Fig. 3E). Then, the three images (Fig. 3C–E) were combined (Fig. 3F). Using Hybrid Cell Count (the quantitative analysis software for the instrument), the numbers of glomerular capillaries and cell nuclei and areas of the glomerular capillaries and glomerular tuft were automatically calculated. These data were used to calculate the cell density per glomerular tuft (in cells/μm^2^) and the capillary area per total glomerular tuft (%).

### Statistical analysis

Statistical tests were performed in Microsoft Excel using BellCurve for Excel (version 3.20; Tokyo, Japan). We assessed differences between the disease groups (control, PSAGN, HSPN, and LN) using the Kruskal–Wallis test and between pairs of groups using the Steel–Dwass test. *P* < 0.05 was considered statistically significant.

## Supplementary Information

Supplementary Information1.

Supplementary Information2.

Supplementary Information3.

Supplementary Information4.

Supplementary Information5.

Supplementary Information6.

## Data Availability

All data generated or analysed during this study are included in this published article (and its Supplementary Information files).
