# Supplementary material for: The severity of glomerular endothelial cell injury is associated with infiltrating macrophage heterogeneity in endocapillary proliferative glomerulonephritis
Source: Sci Rep. 2021 Jun 25;11:13339. doi: 10.1038/s41598-021-92655-5 (PMC8233400; doi:10.1038/s41598-021-92655-5)
Supplement: Supplementary file 1 — Supplementary Information1. [file 41598_2021_92655_MOESM1_ESM.docx]

**Supplementary Information: The severity of glomerular endothelial cell injury is associated with infiltrating macrophage heterogeneity in endocapillary proliferative glomerulonephritis**

Momoko Arai^1^, Akiko Mii^1^, Tetsuya Kashiwagi^1^, Akira Shimizu^2^, Yukinao Sakai^1^

**Supplementary Figure 1. Immunohistochemical detection of glomerular capillaries using several endothelial cell markers**

CD34 immunostaining (A), CD31 immunostaining (B) and vWF immunostaining (C) with PAS counterstain. Scale bars, 50μm.

**Supplementary Figure 2. Endothelial cell proliferation in PSAGN**

Double-immunostaining for CD34 and Ki67 in a patient with PSAGN (A), and a high-magnification image of panel A (B). Arrowhead indicates a double-positive cell. Scale bars, 50μm (A); 20μm (B).

**Supplementary Figure 3. Positive and negative controls for CD3 and CD20 immunostaining of human tonsil tissue.**

Immunohistochemical staining for CD3 (A and C) and CD20 (B and D). Upper panels show positive control. Lower panels show negative control. Scale bars, 200μm.

**Supplementary Figure 4. Distribution of infiltrating macrophages in glomeruli**

Double-immunostaining for type IV collagen (green) and CD68 (red) in PSAGN (A), HSPN (B), and LN (C) samples. Arrowheads indicate infiltrating CD68-positive macrophages into the mesangial and subendothelial areas in glomeruli. Scale bars, 30μm.

**CD206-positive macrophages in glomeruli affected by GN with EP lesions**

CD206 immunostaining in control (A), PSAGN (B), HSPN (C), and LN (D) samples. Arrowheads indicate infiltrating CD206-positive macrophages in glomeruli. Scale bars, 50μm.
