# Supplementary figures and images for: The severity of glomerular endothelial cell injury is associated with infiltrating macrophage heterogeneity in endocapillary proliferative glomerulonephritis
Source: Sci Rep. 2021 Jun 25;11:13339. doi: 10.1038/s41598-021-92655-5 (PMC8233400; doi:10.1038/s41598-021-92655-5)

Supplementary Figure 1

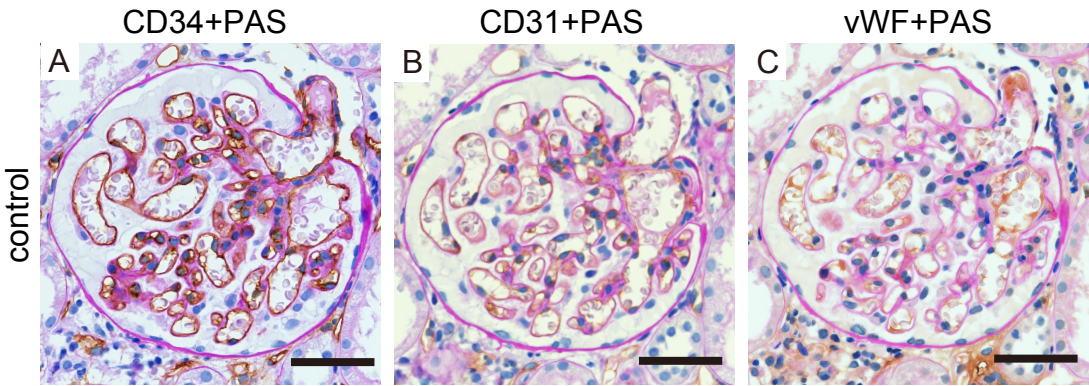

Supplement: Supplementary file 2 — Supplementary Information2. [file 41598_2021_92655_MOESM2_ESM.pdf]

Supplementary Figure 2

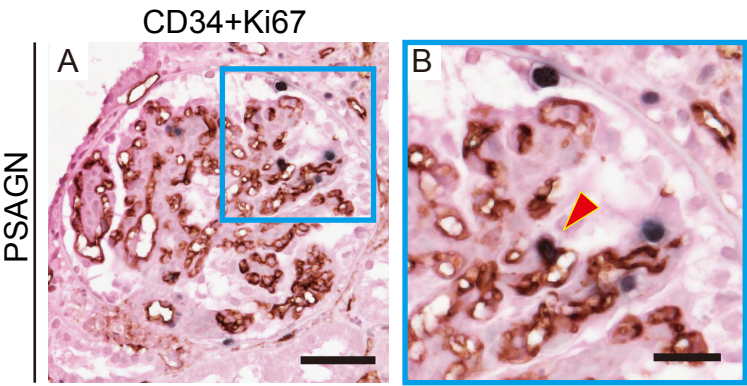

Supplement: Supplementary file 3 — Supplementary Information3. [file 41598_2021_92655_MOESM3_ESM.pdf]

Supplementary Figure 3

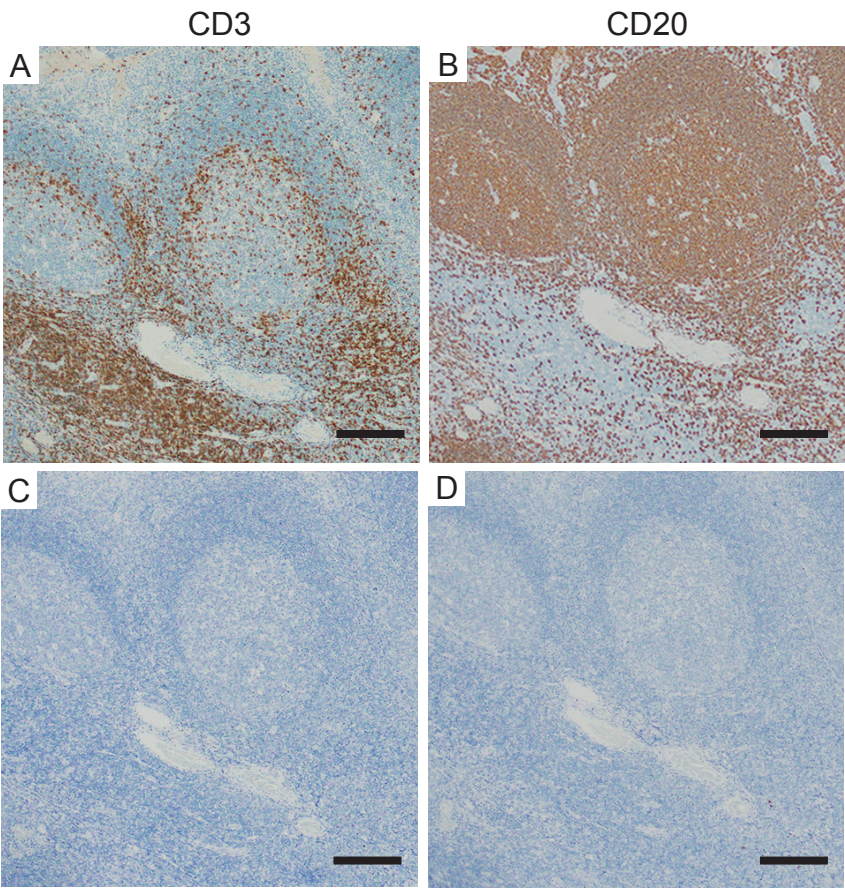

Supplement: Supplementary file 4 — Supplementary Information4. [file 41598_2021_92655_MOESM4_ESM.pdf]

Supplementary Figure 4

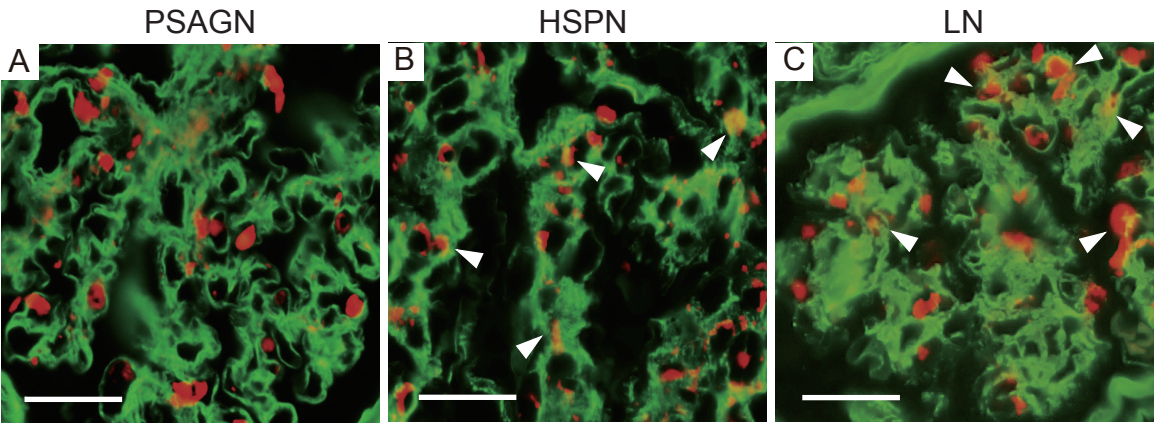

Supplement: Supplementary file 5 — Supplementary Information5. [file 41598_2021_92655_MOESM5_ESM.pdf]

Supplementary Figure 5

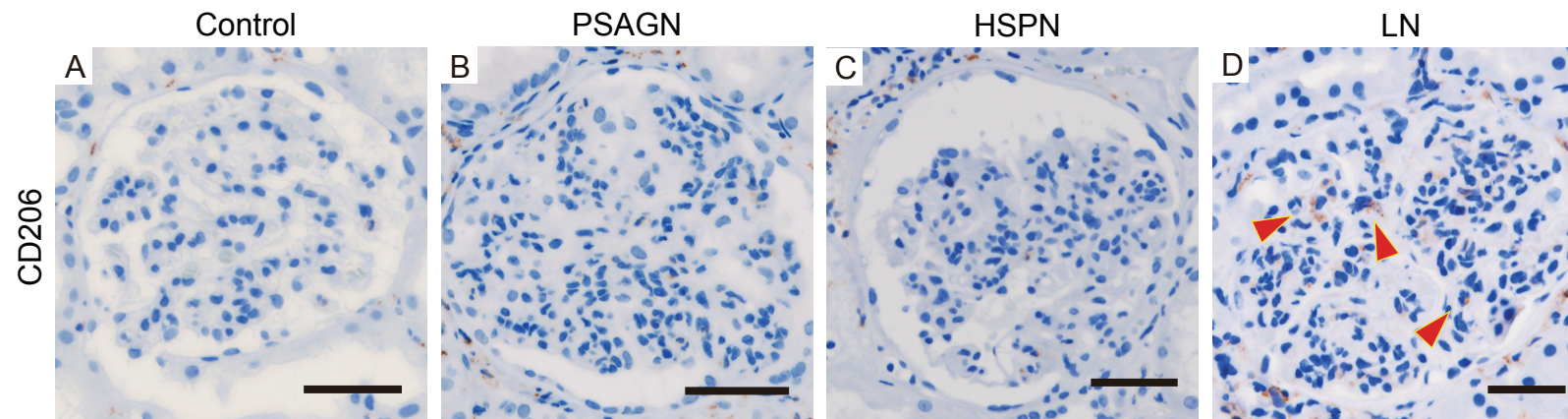

Supplement: Supplementary file 6 — Supplementary Information6. [file 41598_2021_92655_MOESM6_ESM.pdf]
